# Digital media use intensity, cross-border relational ties and generalized social trust: occupational network heterogeneity and neighborhood social embeddedness in contemporary China

**DOI:** 10.3389/fsoc.2026.1911185

**Published:** 2026-10-13

**Authors:** Tuo Zhang, Peng Li, Akmar Hayati Ahmad Ghazali, Nur Atirah Kamaruzaman, Shougui Pang

**Affiliations:** 1 Faculty of Modern Languages and Communication, Universiti Putra Malaysia, Serdang, Malaysia; 2 School of Journalism and Communication, Xi'an International Studies University, Xi'An, China

**Keywords:** cross-border relational ties, digital media use intensity, generalized social trust, local relational embeddedness, relational expansion

## Abstract

Drawing on data from the CGSS 2023 household survey, this study examines how digital media use intensity and cross-border relational ties are associated with generalized social trust through two relational pathways: occupational network heterogeneity and neighborhood social embeddedness. Integrating baseline association models, indirect relational pathway analyses, and robustness checks, this study investigates whether relational expansion and relational embeddedness provide different foundations for generalized trust formation. The results show that digital media use intensity is negatively associated with generalized social trust, whereas cross-border relational ties do not exhibit a significant direct association with generalized social trust. Both digital media use intensity and cross-border relational ties are positively associated with occupational network heterogeneity, indicating broader opportunities for heterogeneous contacts rather than direct evidence of generalized trust formation. However, occupational network heterogeneity is not significantly associated with generalized social trust. In contrast, neighborhood social embeddedness is positively associated with generalized social trust and is involved in a significant indirect association linking digital media use intensity to generalized social trust. Although statistically significant, these associations are modest in magnitude, suggesting that relational conditions explain only a limited proportion of variation in generalized social trust. These findings suggest that the expansion of relational boundaries does not automatically translate into generalized trust. Instead, repeated interaction, mutual support, and locally embedded relational experiences may provide a more stable foundation through which individuals extend trust beyond immediate social circles.

## 1 Introduction

Digital technologies have reshaped the conditions under which individuals organize social relationships and everyday interactions. Rather than functioning merely as channels of information transmission, digital media increasingly participates in the formation and maintenance of social connections. Recent discussions of digital society emphasize that media technologies have become increasingly involved in the processes through which individuals construct, maintain, and experience social relationships, rather than merely serving as neutral communication tools (Couldry and Hepp, 2017; Wang, 2021; Wang et al., 2025). From the perspective of networked social change, digital environments reshape the conditions under which individuals organize communication and social interaction beyond traditional spatial constraints (Castells, 2010).

However, expanded connectivity and increased relational opportunities do not necessarily lead to stronger generalized social trust. Although cooperation among strangers in China has generally increased over time, with urbanization, educational expansion, and internet diffusion identified as important social foundations (Yuan et al., 2024), trust in mediated interactions remains context-dependent and influenced by individuals’ digital practices, social experiences, and existing trust orientations (Ma et al., 2023). This raises an important theoretical question: through which relational pathways do contemporary relational conditions, including digital media use and cross-boundary social connections, become associated with generalized social trust?

The concept of generalized social trust discussed in this study refers to the basic positive expectations that individuals hold toward general others in society, reflecting a generalized orientation regarding whether most people can be regarded as trustworthy beyond immediate personal relationships (Uslaner, 2002; Delhey and Newton, 2005). Unlike particularized trust based on kinship, geographical proximity, and strong ties, generalized trust concerns whether relatively unfamiliar others can be regarded as trustworthy and whether broader social cooperation is possible (Rothstein and Stolle, 2008). As an important dimension of social capital, generalized social trust contributes to public interaction, collective coordination, and the maintenance of social order by enabling individuals to extend expectations of cooperation beyond immediate exchange relationships (Coleman, 1988; Putnam, 2000; Ai and Yuan, 2015).

Meanwhile, contemporary international communication research increasingly recognizes that global connections are experienced not only through institutional communication flows but also through ordinary interpersonal relationships across local and global contexts (Jiang and Zhang, 2022; He and Jiang, 2024). However, such relational connections vary substantially in intensity and content. Based on the above discussion, this study examines how digital media use intensity and cross-border relational ties are associated with generalized social trust through two relational pathways: occupational network heterogeneity and neighborhood social embeddedness. Conceptually, the study distinguishes between two relational orientations.

The focus of this study is therefore not simply whether individuals are connected beyond their immediate social environments, but whether different forms of relational expansion and media use intensity become associated with generalized trust through distinct relational pathways. Based on the China General Social Survey (CGSS) 2023 household survey data, this study addresses three questions: (1) How are digital media use intensity and cross-border relational ties associated with generalized social trust? (2) Through which relational pathways—occupational network heterogeneity or neighborhood social embeddedness—are these associations established? (3) Which relational pathway demonstrates greater empirical consistency with generalized social trust under contemporary Chinese social conditions?

## 2 Literature review and theoretical framework

### 2.1 Generalized social trust: the foundation of social capital and trust extension

In this study, generalized social trust refers to the basic positive expectations that individuals hold toward general others in society, particularly the expectation that people beyond one’s immediate social circles can generally be regarded as trustworthy (Uslaner, 2002; Delhey and Newton, 2005). It differs from particularized trust based on kinship, geographical proximity, and strong ties, as it emphasizes expectations toward relatively unfamiliar others, the possibility of broader social cooperation, and the extension of trust beyond direct interpersonal relationships (Rothstein and Stolle, 2008). Consequently, generalized social trust is regarded as an important foundation for public interaction, cooperation among strangers, and social integration because it enables individuals to extend expectations of cooperation beyond direct exchange relationships (Coleman, 1988; Putnam, 2000; Yang et al., 2020).

Although generalized trust is typically measured as an individual-level orientation, social capital theory emphasizes that trust is closely connected with individuals’ relational experiences and social environments (Coleman, 1988; Putnam, 2000). Individuals do not develop expectations toward broader social others in isolation; rather, such expectations may emerge from accumulated experiences of interaction, cooperation, and social predictability (Yang et al., 2020). Accordingly, generalized trust should not be understood simply as a consequence of having broader relational connections.

From the perspective of trust extension, individuals may generalize experiences from particular relationships to broader social judgments under certain conditions (Uslaner, 2002; Wang and Bian, 2026). However, this process is neither automatic nor linear. Different types of relational experiences may provide different foundations for extending trust beyond immediate social circles. Therefore, understanding generalized social trust requires examining not only whether individuals experience relational expansion or connection opportunities, but also the relational pathways through which such experiences become associated with broader trust orientations.

### 2.2 Relational conditions and relational pathways: digital media use intensity, cross-border relational ties, and network structures

Digital media use intensity and cross-border relational ties represent two contemporary relational conditions through which individuals’ social boundaries may be extended beyond immediate contexts. Rather than treating these factors as direct sources of generalized social trust, this study conceptualizes them as conditions that may shape how individuals form, maintain, and experience social relationships.

Digital media use intensity reflects the increasing integration of digital communication technologies into everyday social life. In the Chinese context, however, digital media practices are embedded within a specific information environment shaped by platform governance, regulatory arrangements, and uneven access to information sources, which may influence how digital engagement is associated with trust orientations. From a digital society perspective, digital media is not merely a channel for information transmission but an infrastructure that reshapes the organization of communication and interpersonal relationships (Couldry and Hepp, 2017). By reducing spatial constraints and lowering communication costs, digital media may provide individuals with additional opportunities to maintain existing relationships and encounter information beyond their immediate social environments (Granovetter, 1973; Wellman et al., 2001; Castells, 2010). However, digital media use does not necessarily generate broader trust orientations. Digital practices may support diverse forms of interaction while simultaneously exposing individuals to uncertain information environments, fragmented communication contexts, and weakly contextualized social encounters (Ji and Zhou, 2017; Chen, 2017). Therefore, the relationship between digital media use intensity and generalized social trust depends on the relational structures and experiences associated with digital practices.

Cross-border relational ties represent another form of relational condition through which individuals’ interpersonal boundaries may extend beyond local contexts. Contemporary communication research increasingly emphasizes that global–local connections are experienced not only through institutional communication or mediated representations but also through everyday interpersonal relationships and ordinary encounters (Jiang and Zhang, 2022; He and Jiang, 2024). From the perspective of social network theory, connections extending beyond immediate relational circles may provide opportunities to encounter heterogeneous others and access diverse social experiences and resources (Granovetter, 1973; Lin, 2001). Similarly, research on transnational networks suggests that cross-border connections may link individuals to social contexts beyond their original communities (Vertovec, 2009; Yang et al., 2015; Huang, 2023).

Cross-border relational ties refer theoretically to interpersonal connections that extend beyond national boundaries. Their significance in the present framework lies in the relational reach they represent rather than in an assumption that such ties necessarily entail intensive transnational communication or produce transnational social capital. Cross-border connections may expose individuals to heterogeneous social experiences, but they may also remain organized within existing family, regional, ethnic, or occupational boundaries (Vertovec, 2009; Duan, 2017; Tan, 2020). Thus, cross-border relational ties represent opportunities for relational boundary extension rather than a direct pathway toward generalized social trust.

Building on this perspective, this study examines two relational pathways through which digital media use intensity and cross-border relational ties may become associated with generalized social trust. The first is occupational network heterogeneity, which represents individuals’ opportunities to encounter socially diverse contacts across occupational boundaries. It captures occupational diversity in relational contacts as a specific dimension of relational expansion and is conceptually distinct from network openness in its broader structural sense. The second is neighborhood social embeddedness, which reflects repeated interaction, relational accessibility, and experiential familiarity within identifiable local relationships. This pathway emphasizes relational embeddedness within specific neighborhood ties rather than generalized expectations toward people beyond those relationships.

### 2.3 Relational pathways and generalized social trust: from social experiences to broader trust orientations

Although relational conditions may expand individuals’ opportunities for social connection, generalized social trust does not emerge automatically from increased connectivity. From the perspective of trust extension, individuals may develop broader expectations toward unfamiliar others by generalizing experiences accumulated within specific relational contexts (Uslaner, 2002). However, social capital theory further suggests that such extension depends on the nature of relational experiences, including whether interactions provide perceptions of reciprocity, predictability, and social reliability (Coleman, 1988; Lin, 2001).

Although connections beyond local contexts may expose individuals to diverse social experiences and unfamiliar social environments, such ties do not necessarily generate generalized trust. Existing cross-border relationships may remain concentrated within family, regional, or identity-based networks and may not involve sustained interaction with socially unfamiliar others (Vertovec, 2009; Duan, 2017). Whether such opportunities become associated with generalized trust depends on the relational structures through which they are organized.

Digital media may facilitate communication across spatial boundaries, maintain weak connections, and provide access to diverse information environments (Wellman et al., 2001; Wang and Zhou, 2019). However, digital interactions may also occur in fragmented or weakly contextualized environments, where increased connectivity does not necessarily produce confidence in broader social others (Han, 2021; Si and Ren, 2025).

Heterogeneous relational connections may expose individuals to people from different occupational and social backgrounds, increasing opportunities for encountering unfamiliar others and accessing diverse perspectives (Granovetter, 1973; Lin, 2001). Such experiences may theoretically reduce social distance and provide opportunities for extending trust beyond immediate circles. However, heterogeneous connections do not necessarily generate generalized trust because diverse contacts may remain superficial, instrumental, or lacking in repeated interaction and reciprocal exchange.

Unlike relational expansion, embedded relationships are characterized by repeated interaction, familiarity, and mutual support within everyday social contexts. Previous research on social capital has emphasized that sustained local interactions can strengthen expectations of reciprocity and social reliability, providing experiential foundations for broader trust orientations (Putnam, 2000; Yang et al., 2020). Crucially, these locally embedded experiences concern relationships with identifiable neighbors and are conceptually distinct from generalized trust, which refers to expectations toward people beyond specific known relationships. Nevertheless, local embeddedness does not automatically produce generalized trust, as locally bounded relationships may remain restricted to particular communities. Its theoretical significance lies in providing repeated experiences through which individuals evaluate the predictability and trustworthiness of others.

### 2.4 Expansion and embeddedness: two relational pathways to generalized social trust

This study distinguishes between two relational pathways: an expansion-oriented pathway represented by occupational network heterogeneity and an embeddedness-oriented pathway represented by neighborhood social embeddedness. This distinction parallels Putnam’s (2000) distinction between bridging and bonding social capital, although the present study does not assume that either form automatically produces generalized trust.

The expansion-oriented pathway emphasizes the extent to which individuals encounter socially heterogeneous connections beyond their immediate relational circles. Therefore, occupational network heterogeneity should be understood as a condition that increases opportunities for relational expansion rather than as a direct source of generalized trust. In contrast, the embeddedness-oriented pathway emphasizes the role of repeated interaction, familiarity, and mutual support within local relational contexts. Neighborhood social embeddedness reflects individuals’ experiences of everyday interaction, relational familiarity, and access to support within identifiable neighborhood relationships. These experiences remain relationally specific, whereas generalized social trust concerns expectations extending beyond identifiable neighborhood ties.

The distinction between these two pathways concerns different relational foundations potentially relevant to trust extension. The expansion-oriented pathway emphasizes opportunities to encounter socially distant others and broaden relational boundaries, whereas the embeddedness-oriented pathway emphasizes repeated interaction, relational familiarity, reciprocity, and predictability within stable social contexts.

Importantly, this distinction does not imply that one pathway is inherently superior to the other. Rather, it recognizes that different relational conditions may provide different foundations for generalized trust. Under contemporary Chinese social conditions, where digital transformation and social mobility continue to reshape interpersonal relationships, it remains an empirical question whether generalized social trust is more consistently associated with expanded relational connections or with embedded relational experiences.

### 2.5 Relational conditions, relational pathways, and generalized social trust: theoretical framework and research hypotheses

Based on the preceding discussion, this study develops an integrated framework to examine how contemporary relational conditions become associated with generalized social trust through different relational pathways.

Rather than assuming that greater connectivity directly produces generalized trust, the study distinguishes between two relational pathways: an expansion-oriented relational pathway represented by occupational network heterogeneity and an embeddedness-oriented relational pathway represented by neighborhood social embeddedness. Accordingly, neighborhood embeddedness and generalized trust are theoretically related but distinct: the former concerns experiences within identifiable local ties, whereas the latter concerns expectations generalized beyond those specific relationships.

Within this framework, digital media use intensity and cross-border relational ties are conceptualized as contemporary relational conditions that may become associated with generalized social trust through different pathways. Digital media use intensity may facilitate relational expansion by reducing communication barriers and enabling broader access to social information, while also supporting locally embedded interactions through everyday communication practices. Therefore, the theoretical focus of this study is not whether contemporary connectivity inherently increases or decreases trust, but rather how different relational conditions become associated with generalized social trust through distinct relational pathways.

Based on this theoretical framework, this study examines four dimensions of association. First, it investigates whether digital media use intensity and cross-border relational ties are associated with generalized social trust. Second, it examines whether these relational conditions are associated with occupational network heterogeneity and neighborhood social embeddedness. Third, it evaluates whether these two relational pathways demonstrate different associations with generalized social trust. Finally, it examines whether occupational network heterogeneity and neighborhood social embeddedness function as indirect relational pathways linking contemporary relational conditions with generalized social trust. Given that the CGSS 2023 data are cross-sectional, the following hypotheses are formulated as theoretically informed associations and indirect relational pathways rather than causal mechanisms or temporal mediation processes. The specific hypotheses are as follows:

H1: Digital media use intensity is negatively associated with generalized social trust.

H2: Cross-border relational ties are associated with generalized social trust.

H3: Digital media use intensity is positively associated with occupational network heterogeneity.

H4: Cross-border relational ties are positively associated with occupational network heterogeneity.

H5: Digital media use intensity is positively associated with neighborhood social embeddedness.

H6: Cross-border relational ties are associated with neighborhood social embeddedness.

H7: Occupational network heterogeneity is positively associated with generalized social trust.

H8: Neighborhood social embeddedness is positively associated with generalized social trust.

H9: Occupational network heterogeneity is indirectly associated with generalized social trust in the relationship between digital media use intensity and generalized social trust.

H10: Neighborhood social embeddedness is indirectly associated with generalized social trust in the relationship between digital media use intensity and generalized social trust.

H11: Occupational network heterogeneity is indirectly associated with generalized social trust in the relationship between cross-border relational ties and generalized social trust.

H12: Neighborhood social embeddedness is indirectly associated with generalized social trust in the relationship between cross-border relational ties and generalized social trust.

Based on the preceding theoretical discussion, this study further compares whether embedded relational experiences demonstrate greater empirical consistency than relational expansion under contemporary Chinese social conditions (see Figure 1).

**Figure 1:**
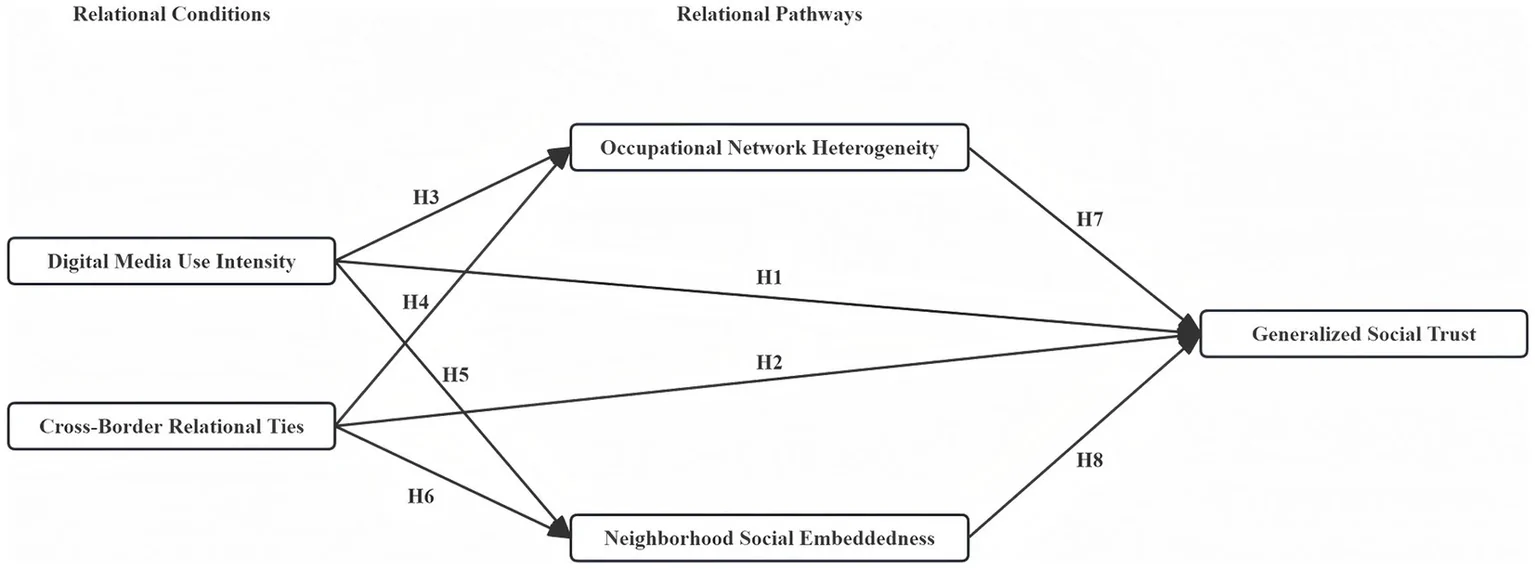
Conceptual framework of relational conditions, relational pathways, and generalized social trust. Solid arrows represent the hypothesized direct associations among digital media use intensity, cross-border relational ties, occupational network heterogeneity, neighborhood social embeddedness, and generalized social trust (H1–H8). Indirect relational pathways through occupational network heterogeneity and neighborhood social embeddedness correspond to H9–H12. The framework also allows for a comparative assessment of the empirical consistency of relational expansion and embedded relational experiences in relation to generalized social trust.

## 3 Research design

### 3.1 Data source and analytical sample

This study uses data from the 2023 Chinese General Social Survey (CGSS), a nationally conducted social survey that collects information on respondents’ sociodemographic characteristics, media use, social relationships, community life, and social attitudes. The CGSS 2023 dataset includes 11,326 respondents, of whom 5,583 participated in the face-to-face household survey and 5,743 in the telephone survey. Because the measures of digital media use intensity, occupational network heterogeneity, and neighborhood social embeddedness are available primarily in the household survey module, the analysis is restricted to the 5,583 household-survey respondents.

A complete-case screening procedure was then applied to the outcome variable, focal explanatory variables, relational pathway variables, and final set of control variables. This yielded an analytical sample of 5,307 respondents, representing 95.1% of the household-survey sample; 276 cases (4.9%) were excluded because of missing information on at least one analytical variable.

All primary models are estimated using this common analytical sample (*N* = 5,307) to ensure comparability across model specifications. Because complete-case analysis may produce selective sample loss, the retained and excluded cases were compared on key sociodemographic characteristics. Excluded respondents were, on average, older, less educated, in poorer self-rated health, of lower subjective socioeconomic status, and more likely to have rural residence or agricultural hukou. These differences suggest that an assumption of data missing completely at random would be difficult to justify. As a sensitivity analysis, 20 imputations were therefore generated for the full household-survey sample using fully conditional specification (FCS); the main substantive patterns remained stable after multiple imputation.

Because the study focuses on examining relational pathways rather than estimating population prevalence parameters, unweighted models are presented as the primary specification. However, given that CGSS provides survey weights designed to adjust sample representativeness, weighted estimates are reported as sensitivity analyses. All data were obtained from the CGSS project administered by the National Survey Research Center at Renmin University of China, and all data processing, model specification, and interpretation were conducted independently by the authors.

### 3.2 Variable operationalization and measurement

This study includes two antecedent variables—digital media use intensity and cross-border relational ties—two relational pathway variables—occupational network heterogeneity and neighborhood social embeddedness—and generalized social trust as the outcome variable. The CGSS indicators used in this study operationalize specific observable dimensions of the broader theoretical constructs developed in Section 2 rather than exhaustive measures of those constructs. Accordingly, each variable is interpreted in terms of the particular empirical dimension captured by the available survey items, and construct-specific boundaries are specified below where necessary. Detailed item sources, operationalization procedures, and coding directions are summarized in Table 1.

**Table 1:** Operationalization and measurement of key variables.

Variable type	Construct/variable	Abbreviation	CGSS Item(s)	Operationalization	Coding/interpretation
Outcome	Generalized social trust	GST	C19	Four-category measure, directionally recoded	Higher = stronger generalized social trust
Antecedent	Digital media use intensity	DMI	A285, A29, A3012	PCA regression component score after directional harmonization and standardization	Higher = greater digital media use intensity
Antecedent	Cross-border relational ties	CBT	C10	Binary indicator	1 = ties present; 0 = no ties
Relational pathway	Occupational network heterogeneity	ONH	C9a–C9j	Sum of 10 occupational-position indicators	0–10; higher = greater occupational heterogeneity
Relational pathway	Neighborhood social embeddedness	NSE	A31a, C13, C14	Single-factor regression score after directional harmonization	Higher = stronger neighborhood social embeddedness
Control	Gender	Female	A2	Binary indicator	1 = female; 0 = male
Control	Age	Age	A3a	Age in years	Higher = older
Control	Education	Edu	A7a	Converted to years of schooling	Higher = more years of education
Control	Hukou status	Hukou	A18	Binary indicator	1 = non-agricultural; 0 = agricultural
Control	Self-rated health	Health	A15	Original five-category scale	Higher = better health
Control	Subjective socioeconomic status	SES	A43e	Directionally recoded	Higher = higher subjective socioeconomic status
Control	Residential area type	Urban	A25a	Binary indicator	1 = urban; 0 = rural

GST is measured using a single CGSS item and is interpreted as an indicator of generalized trust orientation. DMI is based on the first principal component of three internet-use indicators. ONH is a 0–10 count of occupational categories represented in respondents’ relational networks. NSE is a factor-score measure based on three indicators of neighborhood interaction and relational accessibility.

Generalized social trust (GST) was measured using CGSS item C19, which asks whether most people can generally be trusted. Responses were directionally recoded on a four-point scale so that higher values indicate stronger generalized social trust. The item captures respondents’ generalized trust orientation toward people in society rather than trust toward specific identifiable others.

Digital media use intensity (DMI) was constructed from three indicators: frequency of internet use (A285), whether the internet was the respondent’s primary information source (A29), and frequency of internet use during leisure time (A3012). After directional harmonization and standardization, principal component analysis (PCA) was used to extract their common dimension. The Kaiser–Meyer–Olkin (KMO) value was 0.740, and Bartlett’s test of sphericity was significant, *χ*^2^(3) = 10,885.234, *p* < 0.001. Loadings on the first principal component ranged from 0.891 to 0.934, and the component explained 84.093% of the total variance. The regression component score was therefore used as the primary DMI measure.

Cross-border relational ties (CBT) were measured using item C10, which identifies whether respondents had relatives, friends, or acquaintances living abroad. The variable was coded 1 for the presence of such ties and 0 otherwise. CBT therefore indicates the presence of overseas-related relational ties; it does not encode their number, strength, interaction frequency, or communication content.

Following Lin’s (2001) position-generator approach, occupational network heterogeneity (ONH) was constructed from the occupational position-generator items C9a–C9j. Each item indicates whether the respondent knows someone in one of ten occupational categories. Positive responses were coded 1 and summed to form a count measure ranging from 0 to 10, with higher values indicating access to a wider range of occupational positions. The ten occupational categories include university professors, lawyers, nurses, computer programmers, secondary school teachers, human resource managers, farmers, hairdressers, receptionists, and police officers, representing a heterogeneous set of occupational statuses spanning professional, managerial, service, technical, public-sector, and agricultural positions. ONH therefore captures occupational diversity among respondents’ reported relational contacts, which is conceptually distinct from broader structural properties such as network openness or structural holes.

Neighborhood social embeddedness (NSE) was constructed from three indicators: frequency of social interaction with neighbors (A31a), the number of neighbors the respondent greets (C13), and the number of neighbors from whom the respondent could seek help when needed (C14). After directional harmonization, exploratory factor analysis supported a single-factor structure [KMO = 0.579; Bartlett’s *χ*^2^(3) = 2,592.728, *p* < 0.001], with factor loadings of 0.577, 0.830, and 0.845, explaining 57.847% of the total variance. The standardized Cronbach’s *α* was 0.624, and corrected item-total correlations ranged from 0.287 to 0.467, indicating that all retained indicators contributed positively to the construct. Accordingly, the regression factor score was retained as the NSE measure, with higher values representing stronger interaction and relational accessibility within identifiable neighborhood ties. This relationally specific construct is analytically distinguished from GST, which concerns generalized expectations toward people beyond specific known relationships.

The models additionally control for gender, age, education, hukou status, self-rated health, subjective socioeconomic status, and residential area type. Gender was coded 0 = male and 1 = female; age was measured in years; education was converted into years of schooling; hukou status was coded 0 = agricultural and 1 = non-agricultural; higher values of self-rated health and subjective socioeconomic status indicate better health and higher perceived socioeconomic position, respectively; and residential area type was coded 0 = rural and 1 = urban.

### 3.3 Analytical strategy

This study adopts a sequential analytical strategy to examine how digital media use intensity and cross-border relational ties become associated with generalized social trust through different relational pathways. First, descriptive statistics, reliability tests, and correlation analyses are conducted to summarize the distributions of the main variables, evaluate the measurement properties of composite indicators, and examine preliminary associations among focal variables. Given the ordinal nature of generalized social trust, Spearman rank-order correlations are reported as the primary correlation estimates.

Second, although generalized social trust is measured on a four-category ordinal scale, OLS models are used as the primary specification because the outcome categories represent an ordered attitudinal continuum and because coefficient interpretation remains comparable across the sequential models. Ordered and multinomial specifications are additionally estimated as robustness checks. The models progressively introduce control variables, digital media use intensity, cross-border relational ties, occupational network heterogeneity, and neighborhood social embeddedness. These estimates are interpreted as association patterns rather than causal effects because the data are cross-sectional.

Third, to examine whether relational pathways statistically account for part of the associations between contemporary relational conditions and generalized social trust, this study estimates parallel indirect association models using PROCESS Model 4 with 5,000 bootstrap repetitions. Digital media use intensity and cross-border relational ties are modeled separately as antecedent variables, while occupational network heterogeneity and neighborhood social embeddedness are specified as parallel relational pathways. These models estimate indirect associations rather than temporal mediation processes because antecedents, mediators, and outcome were measured in the same survey wave.

Finally, a series of robustness and sensitivity analyses are conducted to evaluate whether the findings are sensitive to alternative estimation strategies, variable constructions, and sample treatment procedures. These analyses include alternative ordinal specifications of generalized social trust, count-model estimations for occupational network heterogeneity, survey-weighted models, alternative measurement of digital media use intensity, multiple imputation procedures, and supplementary interaction analyses examining urban–rural heterogeneity.

Additional diagnostic analyses were conducted to clarify discrepancies between zero-order and covariate-adjusted associations involving neighborhood social embeddedness. For both DMI–NSE and CBT–NSE, covariates were first introduced sequentially in a prespecified cumulative order—gender, age, education, hukou status, self-rated health, subjective socioeconomic status, and urban residence—to trace changes in the focal coefficient across adjustment stages. Separate single-covariate models were also estimated by adding each covariate individually to the focal association. These diagnostics are used to characterize coefficient changes under alternative adjustment sets rather than to identify individual covariates as causal suppressors or explanatory mechanisms. Full results of the DMI–NSE and CBT–NSE diagnostic analyses are reported in Supplementary Tables S2, S3, respectively.

To address potential content overlap between neighborhood social embeddedness and generalized social trust, a targeted measurement sensitivity analysis was additionally conducted. Because GST is measured using a single observed item, conventional latent-variable discriminant-validity procedures are not directly applicable. We therefore constructed a reduced NSE measure excluding C14, the item referring most directly to the availability of help from neighbors. The association between this reduced NSE measure and GST was then re-estimated using the same focal variables and seven covariates as in the primary model. This analysis is intended to assess whether the NSE–GST association is dependent on the help-related item rather than to establish formal latent-variable discriminant validity. Full results of this sensitivity analysis are reported in Supplementary Table S4.

## 4 Empirical results

### 4.1 Measurement assessment, descriptive statistics, and preliminary correlations

Table 2 presents the descriptive statistics of the main variables. The mean level of generalized social trust is 2.890 (SD = 0.731), indicating a moderate level of trust orientation among respondents. Approximately 12% of respondents reported having relatives, friends, or acquaintances living abroad (M = 0.120). The standardized scores of digital media use intensity and neighborhood social embeddedness are centered around zero, consistent with factor-score construction. Occupational network heterogeneity has a mean value of 3.154 (SD = 2.385), suggesting considerable variation in individuals’ access to heterogeneous occupational contacts.

**Table 2:** Descriptive statistics of main variables (*N* = 5,307).

Variable	Mean	SD	Minimum	Maximum
Generalized social trust (GST)	2.890	0.731	1	4
Digital media use intensity (DMI)	0.002	0.999	—	—
Cross-border relational ties (CBT)	0.120	0.324	0	1
Occupational network heterogeneity (ONH)	3.154	2.385	0	10
Neighborhood social embeddedness (NSE)	0.007	0.996	—	—
Female	0.550	0.498	0	1
Age	54.266	16.286	—	—
Education years	9.169	4.511	—	—
Non-agricultural hukou	0.401	0.490	0	1
Self-rated health	3.372	1.130	1	5
Subjective socioeconomic status	2.297	0.879	1	5
Urban residence	0.530	0.499	0	1

DMI and NSE are standardized factor scores. ONH is a count indicator ranging from 0 to 10.

Table 3 reports Spearman correlation coefficients among the core variables. Digital media use intensity is negatively correlated with generalized social trust (*r* = −0.057, *p* < 0.01), whereas neighborhood social embeddedness is positively correlated with generalized social trust (*r* = 0.106, *p* < 0.01). Cross-border relational ties and occupational network heterogeneity do not show significant zero-order associations with generalized social trust. Both digital media use intensity (*r* = 0.286, *p* < 0.01) and cross-border relational ties (*r* = 0.251, *p* < 0.01) are positively associated with occupational network heterogeneity. By contrast, digital media use intensity (*r* = −0.079, *p* < 0.01) and cross-border relational ties (*r* = −0.075, *p* < 0.01) both show negative zero-order correlations with neighborhood social embeddedness. These preliminary associations differ from the covariate-adjusted patterns reported below: the DMI–NSE coefficient becomes positive after adjustment, whereas the negative CBT–NSE association is attenuated to a nonsignificant coefficient. These discrepancies are examined explicitly through supplementary covariate-adjustment diagnostics in Section 4.3.

**Table 3:** Spearman correlations among core variables (*N* = 5,307).

Variable	1	2	3	4	5
1. Generalized social trust (GST)	1				
2. Digital media use intensity (DMI)	−0.057^**^	1			
3. Cross-border relational ties (CBT)	−0.023	0.123^**^	1		
4. Occupational network heterogeneity (ONH)	0.015	0.286^**^	0.251^**^	1	
5. Neighborhood social embeddedness (NSE)	0.106^**^	−0.079^**^	−0.075^**^	0.030^*^	1

Spearman correlations are reported. ^*^p < 0.05, ^**^p < 0.01 (two-tailed tests).

### 4.2 Baseline associations with generalized social trust

Model 1 includes only demographic and socioeconomic controls. After introducing digital media use intensity, cross-border relational ties, occupational network heterogeneity, and neighborhood social embeddedness, Model 2 shows a significant improvement in explanatory power (Δ*R*^2^ = 0.010, *p* < 0.001). As shown in Table 4, digital media use intensity demonstrates a significant negative association with generalized social trust (*B* = −0.049, SE = 0.013, *β* = −0.068, *p* < 0.001). Therefore, H1 is supported. In contrast, cross-border relational ties do not show a significant association with generalized social trust (*B* = −0.034, *p* = 0.292), providing no empirical support for H2.

**Table 4:** Hierarchical OLS models predicting generalized social trust.

Variables	Model 1 B (SE)	Model 2 B (SE)	*β* (Model 2)
Cross-border relational ties (CBT)	—	−0.034 (0.033)	−0.015
Digital media use intensity (DMI)	—	**−0.049 (0.013)**	**−0.068**
Occupational network heterogeneity (ONH)	—	0.006 (0.005)	0.020
Neighborhood social embeddedness (NSE)	—	**0.066 (0.011)**	**0.090**
Female	—	−0.023 (0.020)	−0.016
Age	—	0.001 (0.001)	0.017
Education years	—	0.006 (0.003)	0.035
Non-agricultural hukou	—	−0.016 (0.027)	−0.011
Self-rated health	—	**0.034 (0.010)**	**0.053**
Subjective socioeconomic status	—	**0.034 (0.012)**	**0.041**
Urban residence	—	−0.048 (0.027)	−0.033
*R* ^2^	0.011	0.021	
Adjusted *R*^2^	0.009	0.019	
*F* statistic	8.071^***^	10.199^***^	
Δ*R*^2^	—	0.010***	

*N* = 5,307. Model 1 includes demographic and socioeconomic controls only. Model 2 includes all focal variables and controls. Standard errors are reported in parentheses. ^*^p < 0.05, ^**^p < 0.01, ^***^p < 0.001. Bold values indicate statistically significant estimates.

Occupational network heterogeneity is not significantly associated with generalized social trust (*B* = 0.006, *p* = 0.198), indicating that access to more heterogeneous occupational contacts does not independently correspond to broader trust orientations after controls are introduced. Thus, H7 is not supported. By comparison, neighborhood social embeddedness shows a significant positive association with generalized social trust (*B* = 0.066, SE = 0.011, *β* = 0.090, *p* < 0.001). This result indicates that individuals reporting stronger neighborhood-based interaction and relational accessibility tend to exhibit higher levels of generalized trust orientation. Therefore, H8 is supported.

### 4.3 Indirect relational pathways linking contemporary relational conditions to generalized social trust

Given the cross-sectional nature of the data, these results are interpreted as indirect relational pathways in terms of statistical associations rather than causal mediation effects. As shown in Table 5, digital media use intensity demonstrates significant associations with both relational pathways. Specifically, digital media use intensity is positively associated with occupational network heterogeneity (*B* = 0.1523, *p* < 0.001), suggesting that greater digital media engagement corresponds to broader access to heterogeneous occupational contacts. However, occupational network heterogeneity is not significantly associated with generalized social trust after other variables are considered. Accordingly, the indirect association through occupational network heterogeneity is not significant [Effect = 0.0008, 95% CI (−0.0006, 0.0024)], and H9 is not supported. In contrast, digital media use intensity is positively associated with neighborhood social embeddedness (*B* = 0.0912, *p* < 0.001), and neighborhood social embeddedness is positively associated with generalized social trust. The indirect association through neighborhood social embeddedness is significant [Effect = 0.0061, 95% CI (0.0035, 0.0092)], providing support for H10.

**Table 5:** Parallel indirect relational pathways linking relational conditions to generalized social trust.

Relational condition	Indirect pathway	Effect	95% Bootstrap CI	Hypothesis
Digital media use intensity (DMI)	DMI → ONH → GST	0.0008	[−0.0006, 0.0024]	H9 Not supported
Digital media use intensity (DMI)	DMI → NSE → GST	**0.0061**	**[0.0035, 0.0092]**	**H10 supported**
Cross-border relational ties (CBT)	CBT → ONH → GST	0.0068	[−0.0047, 0.0186]	H11 not supported
Cross-border relational ties (CBT)	CBT → NSE → GST	−0.0010	[−0.0065, 0.0041]	H12 not supported

Bootstrap = 5,000. Confidence intervals excluding zero indicate statistically significant indirect associations. All models control for gender, age, education years, hukou status, self-rated health, subjective socioeconomic status, and urban residence. Because the data are cross-sectional, the estimated pathways represent indirect associations rather than causal mediation effects. The positive DMI → NSE coefficient underlying H10 is covariate-adjusted and reverses the negative zero-order DMI–NSE correlation reported in Table 3. Supplementary sequential and single-covariate diagnostics (Supplementary Table S2) indicate that this sign reversal is conditional on covariate adjustment and is consistent with statistical suppression. Bold values indicate statistically significant indirect associations, as indicated by bootstrap 95% confidence intervals that do not include zero.

Importantly, the positive adjusted association between digital media use intensity and neighborhood social embeddedness should be interpreted in light of its reversal from the zero-order pattern. As shown in Table 3, DMI is negatively correlated with NSE at the bivariate level (*r* = −0.079, *p* < 0.01), whereas the corresponding coefficient becomes positive after adjustment for the demographic and socioeconomic covariates included in the pathway model (*B* = 0.0912, *p* < 0.001). Supplementary sequential and single-covariate diagnostics (Supplementary Table S2) show that, under the prespecified cumulative ordering of covariates, the DMI coefficient first changes from negative to positive when age is introduced (*B* = 0.020, *p* = 0.226) and becomes significantly positive after education is subsequently added (*B* = 0.063, *p* < 0.001). In the single-covariate diagnostics, age alone shifts the coefficient to a small positive but nonsignificant estimate (*B* = 0.018, *p* = 0.261), education alone reduces it essentially to zero (*B* = 0.003, *p* = 0.851), and urban residence substantially attenuates the negative association without reversing its direction (*B* = −0.016, *p* = 0.217). Because coefficient changes across cumulative models depend on the order in which covariates are introduced, these diagnostics should not be interpreted as identifying a single causal suppressor. Rather, they indicate that the sign reversal reflects demographic and socioeconomic composition captured jointly by the covariates, with age and education particularly implicated. The pattern is therefore consistent with statistical suppression, and the positive DMI–NSE association underlying H10 should be understood as conditional on covariate adjustment rather than as an unconditional positive relationship between digital media use and neighborhood embeddedness. Accordingly, the significant indirect association through NSE is interpreted as a small, covariate-adjusted relational pathway rather than as general evidence that greater digital media use strengthens neighborhood embeddedness.

Notably, the direct and indirect associations of digital media use intensity with generalized social trust operate in different directions. The direct association between digital media use intensity and generalized social trust remains negative after including the relational pathways (*B* = −0.0499, *p* < 0.001), whereas the indirect association through neighborhood social embeddedness is positive. This pattern indicates an inconsistent indirect association structure, in which digital media use intensity is simultaneously associated with lower generalized trust directly and with higher generalized trust through locally embedded relational experiences. Thus, the positive indirect pathway should be interpreted as a modest relational channel rather than evidence of a strong mediation process.

Cross-border relational ties are positively associated with occupational network heterogeneity (*B* = 1.2441, *p* < 0.001), indicating that individuals with overseas-related connections tend to report access to a broader range of occupational contacts. This result is further supported by the negative binomial robustness model [IRR = 1.350, 95% CI (1.282, 1.420)]. However, occupational network heterogeneity does not demonstrate a significant association with generalized social trust. Consequently, the indirect association from cross-border relational ties through occupational network heterogeneity is not significant [Effect = 0.0068, 95% CI (−0.0047, 0.0186)], and H11 is not supported.

Cross-border relational ties are not significantly associated with neighborhood social embeddedness (*B* = −0.0166, *p* = 0.687). Accordingly, the indirect association through neighborhood social embeddedness is also not significant [Effect = −0.0010, 95% CI (−0.0065, 0.0041)], providing no support for H12. The total indirect association of cross-border relational ties is likewise non-significant [Effect = 0.0057, 95% CI (−0.0071, 0.0191)]. This adjusted null association differs from the negative zero-order CBT–NSE association reported in Table 3 (*r* = −0.075, *p* < 0.01). Unlike the DMI–NSE pattern, however, the CBT–NSE coefficient does not reverse direction after covariate adjustment; instead, supplementary sequential and single-covariate diagnostics (Supplementary Table S3) show that it is progressively attenuated toward zero. Under the prespecified cumulative adjustment sequence, the coefficient remains significantly negative through adjustment for education (*B* = −0.121, *p* = 0.004) and first becomes nonsignificant after hukou status is subsequently introduced (*B* = −0.051, *p* = 0.227), with further attenuation after urban residence enters the full model (*B* = −0.017, *p* = 0.687). Single-covariate diagnostics further show that urban residence alone reduces the CBT–NSE coefficient to near zero and renders it nonsignificant (*B* = −0.037, *p* = 0.373), whereas education and hukou status individually attenuate but do not eliminate the negative association. Because coefficient changes in cumulative specifications are order-dependent, these results should not be interpreted as identifying hukou status or urban residence as causal explanations. Rather, the diagnostics indicate that the negative zero-order CBT–NSE association partly reflects observed demographic, socioeconomic, and residential composition and does not persist as an independent association after full covariate adjustment.

### 4.4 Robustness and sensitivity analyses

The core robustness and sensitivity results are summarized in Table 6, with additional diagnostic results reported in the Supplementary tables. First, because generalized social trust is measured using an ordinal item, alternative models were estimated using ordered logistic and ordered probit regression. Across these alternative specifications, digital media use intensity remained negatively associated with generalized social trust, whereas neighborhood social embeddedness remained positively associated with generalized social trust. Cross-border relational ties and occupational network heterogeneity did not demonstrate stable associations. However, because the proportional odds assumption was violated in both ordered models, these results are interpreted as supplementary evidence of directional consistency rather than alternative primary estimates. A multinomial logistic specification that relaxes the proportional odds assumption produced similar patterns, with digital media use intensity and neighborhood social embeddedness remaining statistically significant at the model level, while cross-border relational ties and occupational network heterogeneity remained non-significant.

**Table 6:** Robustness and sensitivity checks of core associations.

Robustness test	DMI → GST	NSE → GST	ONH → GST	CBT → GST	Interpretive implication
Baseline OLS	Negative, significant	Positive, significant	Non-significant	Non-significant	Baseline pattern
Ordered logit/probit	Negative, significant	Positive, significant	Non-significant	Non-significant	Supplementary directional consistency
Multinomial logit	Significant	Significant	Non-significant	Non-significant	Robust beyond the proportional-odds specification
Weighted OLS	Negative, significant	Positive, significant	Non-significant	Non-significant	Stable after weighting
Alternative DMI index	Negative, significant	—	—	—	DMI–GST association is not dependent on PCA-based construction
Reduced NSE excluding C14	—	Positive, significant	—	—	NSE–GST association persists after exclusion of the help-related item
Multiple imputation	Negative, significant	Positive, significant	Non-significant	Non-significant	Not driven by missing cases

DMI, digital media use intensity; NSE, neighborhood social embeddedness; ONH, occupational network heterogeneity; CBT, cross-border relational ties. Results summarize the direction and statistical significance of focal associations across alternative model specifications, measurement constructions, weighting procedures, and missing-data treatments. The reduced-NSE specification excludes C14, the item referring to the availability of help from neighbors; detailed results of this sensitivity analysis are reported in Supplementary Table S4. Ordered-model results are interpreted as supplementary directional evidence because the proportional-odds assumption was violated. “—” indicates that the corresponding association was not the focus of that robustness specification.

Second, given that occupational network heterogeneity is constructed as a count indicator, this study further estimated count models using Poisson and negative binomial regression. The negative binomial models show that both digital media use intensity and cross-border relational ties remain positively associated with occupational network heterogeneity. Specifically, digital media use intensity increases the expected occupational network range by approximately 7.5%, while cross-border relational ties increase it by approximately 35.0%.

Third, alternative measurement specifications were examined. Replacing the principal-component-based digital media use intensity indicator with an index constructed by averaging the standardized items produced substantively identical results. Digital media use intensity remained negatively associated with generalized social trust, suggesting that the main finding is not dependent on a specific index construction approach.

Fourth, a targeted measurement sensitivity analysis (Supplementary Table S4) was conducted to assess whether the positive NSE–GST association depended on the help-related C14 item. A reduced NSE measure was constructed from the standardized neighborhood-interaction-frequency item (A31a) and the standardized number-of-neighbors-greeted item (C13), excluding C14. The reduced measure remained strongly correlated with the original three-item NSE index (*r* = 0.895, *p* < 0.001), indicating that it retained much of the empirical content of neighborhood embeddedness. After removing C14, the zero-order association between reduced NSE and GST remained positive and statistically significant (*r* = 0.069, *p* < 0.001). In the fully adjusted model including DMI, CBT, ONH, and the seven demographic and socioeconomic covariates, reduced NSE also remained positively associated with GST [*B* = 0.046, SE = 0.013, *β* = 0.050, *p* = 0.001, 95% CI (0.020, 0.073)]. Compared with the original NSE specification (*β* = 0.090), the association was attenuated but remained statistically significant. Thus, the positive NSE–GST association is not solely attributable to the help-related C14 item. Because GST is measured with a single observed item, however, this sensitivity analysis addresses potential content overlap rather than establishing formal latent-variable discriminant validity.

Fifth, to examine whether the findings were affected by complete-case deletion, this study conducted 20 rounds of fully conditional specification (FCS) multiple imputation using the full household survey sample. The imputed models yielded highly consistent results: digital media use intensity remained negatively associated with generalized social trust, neighborhood social embeddedness remained positively associated, while cross-border relational ties and occupational network heterogeneity remained non-significant.

Sixth, weighted estimates based on CGSS household survey weights were conducted to examine whether sample adjustment influenced the results. The weighted models reproduced the main association patterns, with digital media use intensity showing a negative association and neighborhood social embeddedness showing a positive association with generalized social trust. The positive association between digital media use intensity and neighborhood social embeddedness also remained stable under weighted estimation.

Finally, because multiple indicators were obtained from the same survey instrument, a Harman single-factor diagnostic was conducted as a supplementary assessment of potential common method concerns. The first unrotated factor explained 36.636% of the total variance, below the commonly used 50% reference threshold. However, this diagnostic is limited and cannot fully eliminate concerns regarding common method variance. Therefore, the result is interpreted only as indicating that a single dominant factor does not appear to account for the majority of covariance among the measured variables.

### 4.5 Contextual heterogeneity: urban–rural differences in the associations between relational conditions, relational pathways, and generalized social trust

These analyses are treated as contextual consistency tests rather than causal moderation analyses. As reported in Table 7, the interaction between digital media use intensity and urban residence is statistically significant (*B* = 0.045, *p* = 0.029). Simple slope analyses indicate that DMI is negatively associated with generalized social trust among rural residents (*B* = −0.069, *p* < 0.001), whereas the association becomes statistically insignificant among urban residents (*B* = −0.023, *p* = 0.177). However, the interaction model contributes only limited additional explanatory power (Δ*R*^2^ = 0.001), indicating that urban residence attenuates rather than fundamentally reverses the observed association between digital media use intensity and generalized social trust. The interaction between neighborhood social embeddedness and urban residence is not significant (*B* = −0.004, *p* = 0.863), and neighborhood social embeddedness remains positively associated with generalized social trust among both rural residents (*B* = 0.070, *p* < 0.001), and urban residents (*B* = 0.066, *p* < 0.001).

**Table 7:** Urban–rural heterogeneity in the associations between core relational variables and generalized social trust.

Variables	Rural residents	Urban residents	Urban interaction
Digital media use intensity (DMI)	−0.069^***^	−0.023	0.045*
Cross-border relational ties (CBT)	n.s.	n.s.	n.s.
Occupational network heterogeneity (ONH)	n.s.	n.s.	n.s.
Neighborhood social embeddedness (NSE)	0.070^***^	0.066^***^	−0.004
Model statistics			
Δ*R*^2^			0.001
*F*-change			2.405, *p* = 0.090

Entries are unstandardized coefficients from OLS regression models predicting generalized social trust. All models control for gender, age, education, hukou status, self-rated health, subjective socioeconomic status, and residential area type. Simple slopes are reported for significant interaction terms. ^*^p < 0.05, ^**^p < 0.01, ^***^p < 0.001. n.s., not significant.

Overall, the urban–rural analyses provide supplementary contextual evidence regarding the comparative consistency of different relational pathways. Although the association between digital media use intensity and generalized social trust varies across residential contexts, the positive association between neighborhood social embeddedness and generalized social trust remains stable across rural and urban groups. These findings are consistent with the broader comparative argument developed in this study. Table 8 summarizes the empirical results of all hypotheses.

**Table 8:** Summary of hypothesis testing results.

Hypothesis	Relationship	Result	Decision
H1	DMI → GST (−)	Negative association, *p* < 0.001	Supported
H2	CBT → GST	Not significant	Not supported
H3	DMI → ONH (+)	Positive association, *p* < 0.001	Supported
H4	CBT → ONH (+)	Positive association, *p* < 0.001	Supported
H5	DMI → NSE (+)	Positive covariate-adjusted association, *p* < 0.001; sign reversal from zero-order association	Supported
H6	CBT → NSE	Adjusted association not significant; negative zero-order association attenuated after covariate adjustment	Not supported
H7	ONH → GST (+)	Not significant	Not supported
H8	NSE → GST (+)	Positive association, *p* < 0.001	Supported
H9	DMI → ONH → GST	No significant indirect association	Not supported
H10	DMI → NSE → GST	Significant positive indirect association; conditional on covariate-adjusted DMI–NSE pathway	Supported
H11	CBT → ONH → GST	No significant indirect association	Not supported
H12	CBT → NSE → GST	No significant indirect association	Not supported

## 5 Discussion and conclusions

### 5.1 Discussion and theoretical contributions

This study examined how contemporary relational conditions, including digital media use intensity and cross-border relational ties, become associated with generalized social trust through different relational pathways. Drawing on digital society perspectives, social capital theory, and generalized trust theory, this study moved beyond the assumption that increased connectivity directly produces broader trust orientations. The findings reveal three major patterns.

First, relational expansion indicators examined in this study do not demonstrate a consistent association with generalized social trust. Occupational network heterogeneity itself was not significantly associated with generalized social trust, nor did it constitute a significant indirect pathway linking either digital media use intensity or cross-border relational ties to generalized trust. These findings suggest that occupational diversity among reported contacts alone may be insufficient to generate generalized expectations toward broader social others. Access to socially diverse contacts may expand individuals’ relational boundaries without necessarily involving the repeated interaction, reciprocity, or experiences of social predictability that may be relevant to trust extension. Importantly, this finding pertains specifically to occupational heterogeneity among reported contacts and should not be generalized to network openness or bridging structures more broadly. The results therefore refine existing discussions of relational expansion by showing that expanded relational opportunities and generalized trust orientations remain analytically distinct processes.

Second, neighborhood social embeddedness demonstrates greater empirical consistency than occupational network heterogeneity in its association with generalized social trust. Importantly, this comparative pattern should be interpreted as a difference in empirical consistency rather than evidence that neighborhood embeddedness is universally more important than relational expansion. Unlike occupational network heterogeneity, neighborhood social embeddedness maintained a significant positive association with generalized trust across baseline models, alternative outcome specifications, weighted estimates, multiple imputation analyses, and urban–rural comparisons. The reduced-NSE sensitivity analysis further showed that this positive association persisted after excluding C14, the item most directly referring to the availability of help from neighbors, although the magnitude of the association was attenuated. This result indicates that the observed NSE–GST association is not solely attributable to the help-related item.

The persistence of this association does not imply that neighborhood social embeddedness and generalized social trust are interchangeable constructs. NSE concerns interaction, familiarity, and relational accessibility within identifiable neighborhood ties, whereas GST concerns generalized expectations toward people beyond specific known relationships. From the perspective of generalized trust theory, locally embedded interaction may provide experiential conditions in which expectations of social predictability and reciprocity are encountered or reinforced, while the generalization of such expectations beyond known relationships remains conceptually distinct. The present findings therefore indicate theoretical differentiation combined with empirical association between local embeddedness and generalized trust. Because GST is measured with a single observed item, however, the reduced-NSE analysis addresses potential content overlap rather than establishing formal latent-variable discriminant validity.

Third, digital media use intensity demonstrates a differentiated relational pattern rather than a unidirectional association with generalized social trust. On the one hand, DMI showed a stable negative association with generalized social trust across multiple model specifications. This pattern is consistent with the possibility that intensive digital media use may coexist with informational fragmentation, uneven information credibility, or interactional conditions that do not necessarily foster generalized expectations of social reliability. In the Chinese context, these possibilities may be relevant to understanding the negative DMI–GST association, but the present data do not directly measure media credibility, information disorder, platform governance, or regulatory exposure. They should therefore be treated as theoretically informed interpretations rather than as mechanisms tested by the present analysis.

On the other hand, DMI was positively associated with neighborhood social embeddedness after covariate adjustment, yielding a small positive indirect association with generalized social trust through NSE. This pathway requires particular qualification because the adjusted positive DMI–NSE coefficient reverses the negative zero-order association. The supplementary diagnostics show that, under the prespecified cumulative adjustment sequence, the coefficient first changes direction when age is introduced and becomes significantly positive after education is subsequently added; single-covariate analyses likewise indicate substantial sensitivity to age, education, and urban residence. Because these coefficient changes depend on the covariate specification and, for cumulative models, the order of adjustment, they should not be interpreted as identifying a single causal suppressor. Rather, the pattern indicates that the positive DMI–NSE association is conditional on demographic and socioeconomic adjustment. H10 should therefore be understood as a small, covariate-adjusted and model-dependent indirect relational pathway rather than as evidence that greater digital media use generally strengthens neighborhood embeddedness.

The coexistence of a negative direct DMI–GST association and a positive indirect association through NSE further constitutes an inconsistent indirect association structure. This pattern suggests that digital media engagement may be associated with generalized trust through relational and informational processes operating in different directions. Digital media should therefore not be understood as either a uniformly trust-enhancing or trust-reducing force. Instead, it represents a contemporary relational condition whose association with generalized trust varies across the relational contexts with which digital practices are connected.

The findings for cross-border relational ties provide a further qualification to the relationship between relational reach and local embeddedness. Although CBT showed a negative zero-order association with NSE, this association was progressively attenuated toward zero after covariate adjustment and did not reverse direction. The supplementary diagnostics indicate that the bivariate relationship is sensitive to the demographic, socioeconomic, and residential composition associated with cross-border ties, with urban residence alone substantially reducing the association. Because these patterns are adjustment-dependent, they do not support an interpretation in which cross-border ties independently weaken neighborhood embeddedness. Rather, the findings suggest that the negative zero-order association partly reflects the social and residential locations in which cross-border relational resources are distributed. More broadly, CBT is strongly associated with occupational network heterogeneity but does not exhibit a significant indirect association with generalized trust through either relational pathway. Cross-border relational reach and generalized trust therefore appear to represent distinct relational dimensions in the present data.

These findings contribute to existing research in three ways. First, this study extends explanations of generalized social trust beyond a connectivity-centered perspective. By distinguishing relational conditions from relational pathways, this study demonstrates that connectivity itself does not determine trust outcomes. Instead, the relational experiences generated through different forms of connection are central to understanding how generalized trust becomes associated with contemporary social change.

Second, this study contributes to social capital research by distinguishing between expansion-oriented and embeddedness-oriented relational pathways. Building on the distinction between bonding and bridging social capital (Putnam, 2000), this study does not treat occupational network heterogeneity as an equivalent measure of bridging capital, but instead examines it as an empirical indicator of relational expansion. Similarly, neighborhood social embeddedness captures locally embedded relational experiences that partially correspond to bonding-type processes. This distinction allows the study to examine how different relational structures are associated with generalized trust without assuming that bridging or bonding orientations automatically generate trust.

Third, this study advances communication research by conceptualizing digital media use intensity as a relational condition rather than merely as a technological exposure variable. Digital media use is not associated with generalized trust simply through access or frequency of use; rather, it is connected with different relational opportunities and contexts through which individuals maintain relationships, encounter others, and interpret social experiences. The coexistence of a negative DMI–GST association and a small, covariate-adjusted positive indirect association through neighborhood embeddedness further indicates that the relationship between digital communication and generalized trust cannot be reduced to a single connectivity effect. By linking digital media practices with differentiated relational pathways, this study provides a relational perspective on how communication environments are associated with broader social trust orientations.

These findings also bear on debates concerning media governance and the public sphere. The negative association between digital media use intensity and generalized social trust suggests that greater digital participation should not be assumed automatically to strengthen the social foundations of public communication. From a media-governance perspective, the relational and informational conditions under which mediated communication occurs—including information credibility, interaction quality, and the connection between mediated communication and everyday social relations—may be relevant to generalized expectations toward unfamiliar others. This implication is particularly pertinent to the public sphere, where communication across social boundaries depends not only on access to media but also on expectations of social reliability beyond immediate personal networks. At the same time, the comparatively consistent association between neighborhood social embeddedness and generalized trust suggests that digitally mediated public communication should be considered in relation to the offline relational environments in which individuals remain embedded. Because the present study does not directly measure platform governance, regulatory exposure, content moderation, or media-policy interventions, these implications should be understood as theoretically informed extensions of the findings rather than as tested effects of media governance.

### 5.2 Conclusion

This study investigated how digital media use intensity and cross-border relational ties become associated with generalized social trust through different relational pathways in contemporary China. The findings demonstrate that contemporary connectivity does not automatically translate into broader trust orientations. Three conclusions can be drawn. First, digital media use intensity is negatively associated with generalized social trust, while simultaneously showing a small positive indirect association through neighborhood social embeddedness that emerges after covariate adjustment. This indicates that digital media practices may participate in multiple relational processes with different implications for trust, although the indirect pathway should be interpreted cautiously because its first-stage association reverses the negative zero-order DMI–NSE relationship and is therefore conditional on covariate adjustment rather than reflecting an unconditional positive DMI–NSE association.

Second, cross-border relational ties and digital media use intensity are associated with broader occupational network heterogeneity, but such relational expansion does not demonstrate a stable association with generalized social trust. The negative zero-order association between cross-border relational ties and neighborhood social embeddedness is likewise attenuated to a nonsignificant association after covariate adjustment, indicating that this bivariate relationship does not persist independently of observed demographic, socioeconomic, and residential composition.

Third, neighborhood social embeddedness demonstrates a more consistent association with generalized social trust than occupational network heterogeneity across different model specifications, weighting procedures, missing-data treatments, urban–rural contexts, and the reduced-item sensitivity analysis. These findings are consistent with the possibility that accumulated experiences of interaction, reciprocity, and social predictability provide a more empirically consistent relational context for generalized trust than relational expansion alone. Overall, this study argues that generalized social trust should not be understood as a direct outcome of increasing connectivity. Instead, the findings indicate that generalized trust is differentially associated with the relational pathways through which individuals experience, interpret, and extend expectations toward broader social others.

### 5.3 Limitations and future research

Several limitations should be acknowledged. First, digital media use intensity captures the extent of digital media incorporation into everyday life but does not distinguish among different types of digital practices. Cross-border relational ties identify the existence of overseas-related connections but do not measure interaction frequency, relationship strength, or communication content. In addition, generalized social trust is measured using a single survey item. Although this item is widely used in trust research, it cannot capture the full complexity of generalized trust orientations.

Relatedly, although neighborhood social embeddedness and generalized social trust are theoretically distinguished by the specificity of their relational referents, some conceptual proximity remains possible because one NSE item refers to the availability of help from neighbors. The reduced-NSE sensitivity analysis shows that the positive NSE–GST association persists after this item is removed, indicating that the association is not solely attributable to the help-related item. However, because GST is measured using a single observed item, the present study cannot conduct a formal latent-variable assessment of discriminant validity. Future research using multi-item measures of generalized and particularized trust could examine this distinction more directly.

Second, this study relies on cross-sectional survey data. Although the analysis identifies theoretically informed association patterns and indirect relational pathways, it cannot establish temporal ordering or causal mechanisms. Future longitudinal research could further examine how relational experiences accumulate over time and contribute to changes in generalized trust orientations. Moreover, the negative association between digital media use intensity and generalized trust may reflect selection processes, such as individuals with lower prior trust orientations adopting different patterns of media use. Similarly, the positive association between neighborhood social embeddedness and generalized trust does not establish whether embedded relational experiences precede generalized trust or whether prior trust orientations facilitate neighborhood interaction. Future research using panel data, longitudinal designs, or stronger identification strategies could further examine the temporal relationship between media practices and trust development.

Third, because all variables were obtained from the same survey wave and primarily relied on self-reported measures, common method variance cannot be fully excluded. Although diagnostic analyses did not indicate a dominant single-factor structure, future studies combining survey data with behavioral indicators or longitudinal information could further reduce this concern. Future research should also examine how institutional features of digital media environments, including platform governance, information regulation, and perceived media credibility, shape the relationship between digital practices and generalized trust. Such research would provide a more direct basis for evaluating how media-governance arrangements influence the relational foundations of communication within the public sphere.

## Data Availability

The data analyzed in this study is subject to the following licenses/restrictions: Raw data collected and analyzed in this study are available from Chinese General Social Survey (CGSS): https://www.cnsda.org/index.php. CGSS was conducted by the National Survey Research Centre at Renmin University of China. According to the data usage license agreement, these materials are not publicly available. However, the researcher may obtain CGSS data by submitting a formal application to the survey management team. All data requests must comply with the CGSS terms of use and confidentiality provisions. Requests to access these datasets should be directed to https://www.cnsda.org/index.php.
