# Supplementary material for: Digital media use intensity, cross-border relational ties and generalized social trust: occupational network heterogeneity and neighborhood social embeddedness in contemporary China
Source: Front Sociol. 2026 Oct 13;11:1911185. doi: 10.3389/fsoc.2026.1911185 (PMC13710036; doi:10.3389/fsoc.2026.1911185)
Supplement: Supplementary file 1 [file Supplementary_file_1.docx]

Supplementary Material

# Table S1. Multicollinearity Diagnostics for Regression Predictors

| **Predictor** | **Tolerance** | **Variance Inflation Factor (VIF)** |
| --- | --- | --- |
| Digital media use intensity (DMI) | .629 | 1.591 |
| Neighborhood social embeddedness (NSE) | .851 | 1.175 |
| Cross-border relational ties (CBT) | .886 | 1.129 |
| Occupational network heterogeneity (ONH) | .753 | 1.329 |
| Gender (Female = 1) | .958 | 1.044 |
| Age | .524 | 1.909 |
| Years of Education | .543 | 1.842 |
| Urban Household Registration | .546 | 1.833 |
| Self-rated Health | .814 | 1.228 |
| Subjective Socioeconomic Status (SES) | .922 | 1.084 |
| Urban Residence | .535 | 1.869 |
| **Note.** VIF values were calculated based on the full regression specification including all focal predictors and control variables. Following conventional criteria, VIF values below 5 indicate that multicollinearity is unlikely to pose a serious concern. In the present study, all predictors showed acceptable levels of collinearity, with VIF values ranging from 1.044 to 1.909. | | |

**Supplementary Table S2. Sequential and Single-Covariate Diagnostics for the Association between Digital Media Use Intensity and Neighborhood Social Embeddedness**

| **Panel A. Sequential cumulative adjustment** | | | | | | |
| --- | --- | --- | --- | --- | --- | --- |
| **Model** | **Covariate added** | **DMI B** | **SE** | **β** | **p** | **95% CI** |
| M0 | None | −.078 | .014 | −.079 | <.001 | [−.105, −.052] |
| M1 | + Gender | −.076 | .014 | −.076 | <.001 | [−.103, −.049] |
| M2 | + Age | .020 | .016 | .020 | .226 | [−.012, .051] |
| M3 | + Education | .063 | .017 | .064 | <.001 | [.031, .096] |
| M4 | + Hukou | .081 | .016 | .082 | <.001 | [.049, .114] |
| M5 | + Health | .078 | .017 | .078 | <.001 | [.046, .111] |
| M6 | + SES | .078 | .016 | .079 | <.001 | [.046, .111] |
| M7 | + Urban residence | .091 | .016 | .091 | <.001 | [.060, .123] |
| **Panel B. Single-covariate diagnostics** | | | | | | |
| **Model** | **Adjustment** | **DMI B** | **SE** | **β** | **p** |  |
| Base | None | −.078 | .014 | −.079 | <.001 |  |
| 1 | Gender only | −.076 | .014 | −.076 | <.001 |  |
| 2 | Age only | .018 | .016 | .018 | .261 |  |
| 3 | Education only | .003 | .015 | .003 | .851 |  |
| 4 | Hukou only | −.055 | .014 | −.055 | <.001 |  |
| 5 | Health only | −.070 | .014 | −.070 | <.001 |  |
| 6 | SES only | −.080 | .014 | −.080 | <.001 |  |
| 7 | Urban residence only | −.016 | .013 | −.017 | .217 |  |
| **Note.** N = 5,307 in all models. The dependent variable is neighborhood social embeddedness (NSE). Panel A introduces covariates cumulatively in the prespecified order shown; each model therefore retains all covariates introduced in preceding models. Panel B adds each covariate separately to the unadjusted DMI–NSE specification. Because coefficient changes in cumulative models depend on covariate ordering, these diagnostics are descriptive of the adjustment pattern and should not be interpreted as identifying a single causal suppressor. B denotes the unstandardized regression coefficient, SE the standard error, and β the standardized regression coefficient. All models are estimated using the common complete-case analytical sample employed in the primary analyses. *p* < .05, *p* < .01, *p* < .001. | | | | | | |

**Supplementary Table S3. Sequential and Single-Covariate Diagnostics for the Association between Cross-Border Relational Ties and Neighborhood Social Embeddedness**

| Panel A | | | | | |
| --- | --- | --- | --- | --- | --- |
| **Model** | **Covariate added** | **CBT B** | **SE** | **β** | **p** |
| M0 | None | −.229 | .042 | −.075 | <.001 |
| M1 | + Gender | −.226 | .042 | −.074 | <.001 |
| M2 | + Age | −.201 | .042 | −.065 | <.001 |
| M3 | + Education | −.121 | .043 | −.039 | .004 |
| M4 | + Hukou | −.051 | .042 | −.017 | .227 |
| M5 | + Health | −.048 | .042 | −.016 | .252 |
| M6 | + SES | −.056 | .042 | −.018 | .181 |
| M7 | + Urban residence | −.017 | .041 | −.005 | .687 |
| **Panel B** | | | | | |
| **Model** | **Adjustment** | **CBT B** | **SE** | **β** | **p** |
| Base | None | −.229 | .042 | −.075 | <.001 |
| 1 | Gender only | −.226 | .042 | −.074 | <.001 |
| 2 | Age only | −.204 | .042 | −.066 | <.001 |
| 3 | Education only | −.108 | .043 | −.035 | .011 |
| 4 | Hukou only | −.104 | .042 | −.034 | .014 |
| 5 | Health only | −.225 | .042 | −.073 | <.001 |
| 6 | SES only | −.241 | .042 | −.078 | <.001 |
| 7 | Urban residence only | −.037 | .041 | −.012 | .373 |
| **Note.** N = 5,307 in all models. The dependent variable is neighborhood social embeddedness (NSE), and cross-border relational ties (CBT) is the focal predictor. Panel A reports sequential cumulative-adjustment models. Model 0 includes CBT only; Models 1–7 introduce gender, age, education, hukou status, self-rated health, subjective socioeconomic status, and urban residence cumulatively in the order shown, with each model retaining all covariates introduced in preceding models. Panel B reports single-covariate diagnostic models in which each covariate is added separately to the unadjusted CBT–NSE specification. B denotes the unstandardized regression coefficient, SE the standard error, and β the standardized regression coefficient. All models are estimated using the common complete-case analytical sample employed in the primary analyses. Because changes in the CBT coefficient across cumulative specifications depend partly on the order in which covariates are introduced, these diagnostics are intended to characterize the attenuation of the zero-order CBT–NSE association under alternative adjustment sets rather than to identify any individual covariate as a causal explanation of that association. *p* < .05, *p* < .01, *p* < .001. | | | | | |

**Supplementary Table S4. Sensitivity Analysis of the Association between Reduced Neighborhood Social Embeddedness and Generalized Social Trust**

| **Specification** | **Association** | **B** | **SE** | **β / r** | **p** | **95% CI** |
| --- | --- | --- | --- | --- | --- | --- |
| Original NSE | Zero-order correlation with GST | — | — | r = .106 | <.001 | — |
| Reduced NSE | Zero-order correlation with GST | — | — | r = .069 | <.001 | — |
| Original NSE | Fully adjusted association with GST | .066 | .011 | β = .090 | <.001 | — |
| Reduced NSE | Fully adjusted association with GST | .046 | .013 | β = .050 | .001 | [.020, .073] |
| **Note.** Reduced NSE excludes C14, the item referring to the availability of help from neighbors, and is constructed as the mean of standardized A31a and C13. The reduced measure is strongly correlated with the original three-item NSE index (r = .895, p < .001). Fully adjusted models include DMI, CBT, ONH, gender, age, education, hukou status, self-rated health, subjective socioeconomic status, and urban residence. N = 5,307. Because GST is measured using a single observed item, this analysis is interpreted as a sensitivity assessment of potential content overlap rather than a formal latent-variable test of discriminant validity. | | | | | | |
